# Wave-breaking modulation by infragravity waves during an extreme typhoon

**DOI:** 10.1371/journal.pone.0231242

**Published:** 2020-04-14

**Authors:** Yoshinao Matsuba, Takenori Shimozono, Shinji Sato

**Affiliations:** 1 Department of Civil Engineering, University of Tokyo, Tokyo, Japan; 2 School of Systems Engineering, Kochi University of Technology, Kochi, Japan; Institute of Oceanology Chinese Academy of Sciences, CHINA

## Abstract

Herein, we present coastal-wave records under historically extreme conditions caused by a strong typhoon in Japan in 2017. The extreme typhoon generated large infragravity waves, reaching a height of 2 m in shallow water. We took advantage of the extraordinary conditions to analyze the effect of the energetic infragravity waves on the nearshore evolution of relatively short waves. Individual wave analyses clearly demonstrate that the instantaneous water-level rise and drawdown caused by the infragravity waves alternately decelerated and accelerated the breaking of short waves under extreme conditions. This mechanism transmitted the large short-wave energy on the infragravity wave crests to the shore, eventually increasing the height of the nearshore waves. This study provides *in situ* evidence that the infragravity waves significantly affect nearshore wave characteristics under extreme conditions.

## 1. Introduction

Waves play a dominant role in determining nearshore environment characteristics, such as nearshore circulation, related morphological changes, and water quality. High wave runups can significantly impact coastal communities, causing flooding, destruction of coastal and harbor structures, and morphological changes at the beach, especially under extreme conditions caused by tropical cyclones or other severe storms. As a recent example, we can consider the Super Typhoon Haiyan, which caused severe flooding of reef-lined coasts in the Philippines along with an extreme wave runup in 2013 [1,2]. More recently, Hurricanes Irma and Maria devastated extensive coastal areas of several Caribbean Islands in 2017 [3,4]. During such extreme conditions, waves strongly interact with themselves and with currents, while the resulting complex wave fields and morphological changes cause significant impacts on nearshore areas. Therefore, it is crucial to understand the extreme nearshore dynamics.

Especially under extreme conditions, wave nonlinearity increases in nearshore areas, where the wave height becomes comparable with the local water depth. This increase in wave nonlinearity affects the irregular nearshore wave fields in a complex manner. Incident short (SS) waves (>0.04 Hz) generally excite their superharmonic components while propagating to the shore. The SS waves eventually dissipate the majority of their energy through wave breaking, which caps amplification. Therefore, wave runup at the shore tends to become saturated. However, the nonlinear interactions among different-frequency components excite the subharmonic (long-wave) components, which can be referred to as infragravity (IG) waves (0.005–0.04 Hz) [5]. The IG waves are excited as bound waves by spatially varying the radiation stress after the grouping of the incident irregular waves [6]. The bound waves are released as free waves through SS-wave breaking and/or the decrease of wave dispersion in shallow water [7]. IG waves are also considered to be excited by breaking-point oscillations [8] and are known to dissipate some of their energy and cause runup saturation [9–12] by transferring energy to the SS waves [13,14] or to their superharmonics [15–17]. However, when excited by energetic offshore forcing, the IG waves may reach heights on the order of 1 m, thereby becoming a significant factor in nearshore wave dynamics [18–20].

IG waves significantly affect nearshore wave dynamics because the magnitude of the IG-wave-frequency motion becomes comparable to water depth and the magnitude of SS waves after the decrease of the water depth. IG waves’ dominance over SS waves during wave runup has been shown in some previous studies, such as during Typhoon Haiyan along the reef-lined coasts [2,21,22]. The IG waves can also accelerate and decelerate the SS waves because of the instantaneous water-level change and currents caused by the IG waves over uniformly sloping beaches [23]. SS waves modulated by IG waves are considered to be a key nearshore feature under conditions of extreme dynamics. However, studies on combined SS and IG waves under extreme conditions are rare because such conditions cannot be completely reproduced using numerical simulations or laboratory experiments. Elucidation requires *in situ* observation, but it is challenging to perform field measurements under such rare and extreme conditions.

Herein, we present novel observations of the extreme coastal waves that occurred in Japan after Typhoon Lan in 2017. Two bottom-pressure sensors that were deployed on a high-wave-prone coast survived the extreme conditions in which the significant wave height exceeded 8.0 m during the passage of the typhoon. The IG waves reached a height of approximately 2.0 m, significantly modulating the SS waves in shallow water. We took advantage of these extraordinary conditions, including large IG waves, to study the characteristics of the IG waves and their effect on the evolution of SS waves from relatively deep water to nearshore areas.

## 2. Field observation

### 2.1 Typhoon Lan and field site

**Fig 1:**
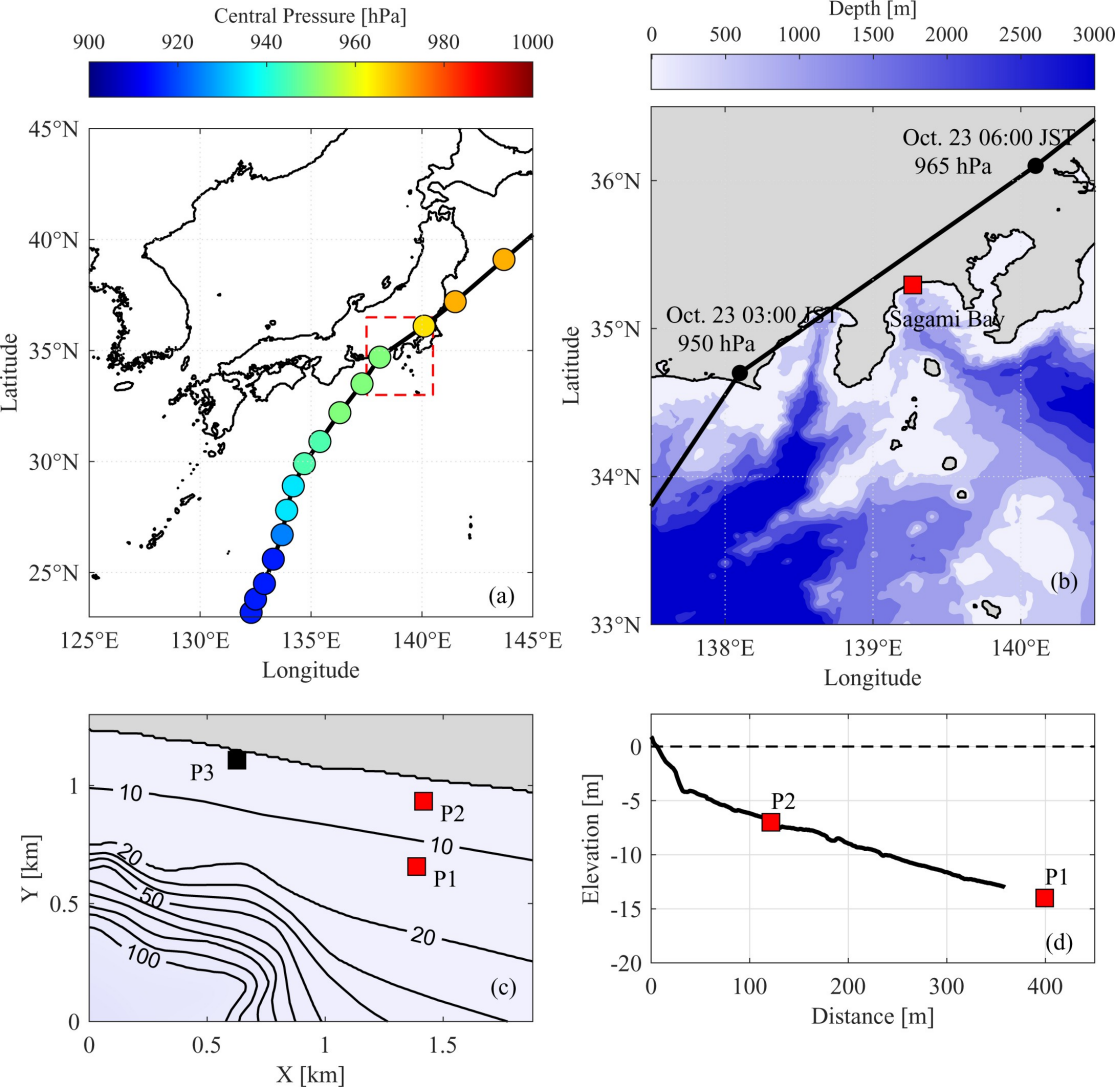
(a) Track of Typhoon Lan showing central pressure (measured at 3-hr intervals by the Japan Meteorological Agency), with the red rectangle outlining the study area. (b) Map of study area showing segment of typhoon track and location of specific target site (red square); bathymetry (30 arc-second data), is from general bathymetric chart of the oceans (GEBCO). (c) Map of measurement points (contour interval, 10 m). P1 and P2 are locations of bottom-pressure sensors; P3 is location of nearshore video camera. (d) Cross-shore topography along transect obtained in 2015 from wave-gage locations.

In the early morning of October 23, 2017, a category-4 typhoon called Typhoon Lan, exhibiting a lowest center pressure of 915 hPa and a maximum wind velocity of 50 m/s, made landfall in Shizuoka Prefecture in Japan (Fig 1(A) and 1(B)). The typhoon was categorized as a super-large typhoon by the Japan Meteorological Agency owing to its wide and strong wind field (wind speeds exceeding 15 m/s at a distance of 800 km from the center of the typhoon). Typhoon Lan, the first super-large typhoon on record to make landfall in Japan since 1991, caused severe damage due to high waves, strong winds, and heavy rains in almost the entire country.

The target site in this study, the Seisho coast, which is at the head of the Sagami Bay, is characterized by steep terrain and deep bathymetry along the Sagami Trough (Fig 1(B)). The Seisho coast has often been adversely affected by high-wave attacks because of its steep coastal topography, where water depth is 100 m at an offshore distance of 1 km. A notable case is that of Typhoon Fitow (in 2007), in which an expressway running along the coast was extensively damaged by high waves. In a previous study, Tajima and Sato [24] suggested that the associated IG waves played a key role in that serious coastal damage. The same vulnerable coast was severely damaged again due to the extreme wave fields during the passage of Typhoon Lan. Two bottom-pressure sensors and one surveillance camera, deployed on the coast by the Japan Ministry of Land, Infrastructure, Transport, and Tourism, survived the severe wave and wind conditions while keeping records of the extreme nearshore waves. Observed time series of nearshore water level during an extreme typhoon are especially valuable because wave observation under such severe conditions is challenging. We analyzed the wave data to understand the effects of IG waves on the extreme nearshore wave field.

### 2.2 Wave data processing

The bottom-pressure data during the typhoon passage were provided by the Japan Ministry of Land, Infrastructure, Transport, and Tourism. The bottom pressure was measured for 20 min every hour at 2 Hz, by two pressure sensors (WAVE HUNTER; IOTechnic) installed along a cross-shore transect at P1 and P2 (Fig 1(C)). The wave-pressure sensors at P1 and P2 started the 20-minute observation periods at 10 minutes before and on the hour, respectively. Pressure sensor gage (P1) was ~400 m offshore at a depth of 14 m, whereas the onshore wave gage (P2) was ~120 m offshore at a depth of 6 m (Fig 1(D)). The water level was estimated with a 0.2 Hz cutoff frequency. The conversion factor of each frequency component of pressure head to water level was estimated according to the Airy wave theory. Some estimation error can arise from the choice of cutoff frequency and deviation from Airy wave theory. The sensor measurement resolution of the pressure head is ~0.01 m, and the 0.2 Hz cutoff frequency was chosen such that the conversion factor from pressure head to water level did not exceed 5 at P1. Deviation from Airy wave theory cannot be determined, but previous case studies suggested that the error of wave-height estimation from bottom-pressure data is at most 10% with a proper cutoff frequency [25]. The both errors might apply when dealing with relatively high-frequency components, but the dominant SS components of the present case are swells of relatively low frequency (<0.1 Hz). Therefore, the error is expected to be negligible. Note that we could not correct for the effects of air-pressure oscillations due to the lack of onsite atmospheric-pressure data, but the IG frequency oscillations in atmospheric-pressure were assumed to be sufficiently small, given that a height of the observed storm surge at Odawara along the Seisho coast was less than 1 m (according to the Japan Meteorological Agency).

We also used videos captured by a surveillance camera (854 × 480; 30 fps) installed at P3, 1 km west of the transect (Fig 1(C)), to observe the waves in the nearshore area. From 05:30 to 12:30 JST on October 23, the camera captured seven 1-hr-long videos of the nearshore area at a depth of 1–2 m. The variation in water level during this period was extracted from time-stack images of the water surface in front of a breakwater (S1 Appendix). The resulting change in mean water level agreed well with the observed water-level data at a nearby tide station (root-mean-squared-error 0.03 m). Although P3 is offset along the shore from P1 and P2, the observed wave data are assumed to represent the nearshore wave statistics for similar incident waves.

## 3 Analysis

### 3.1 Observed wave characteristics

**Fig 2:**
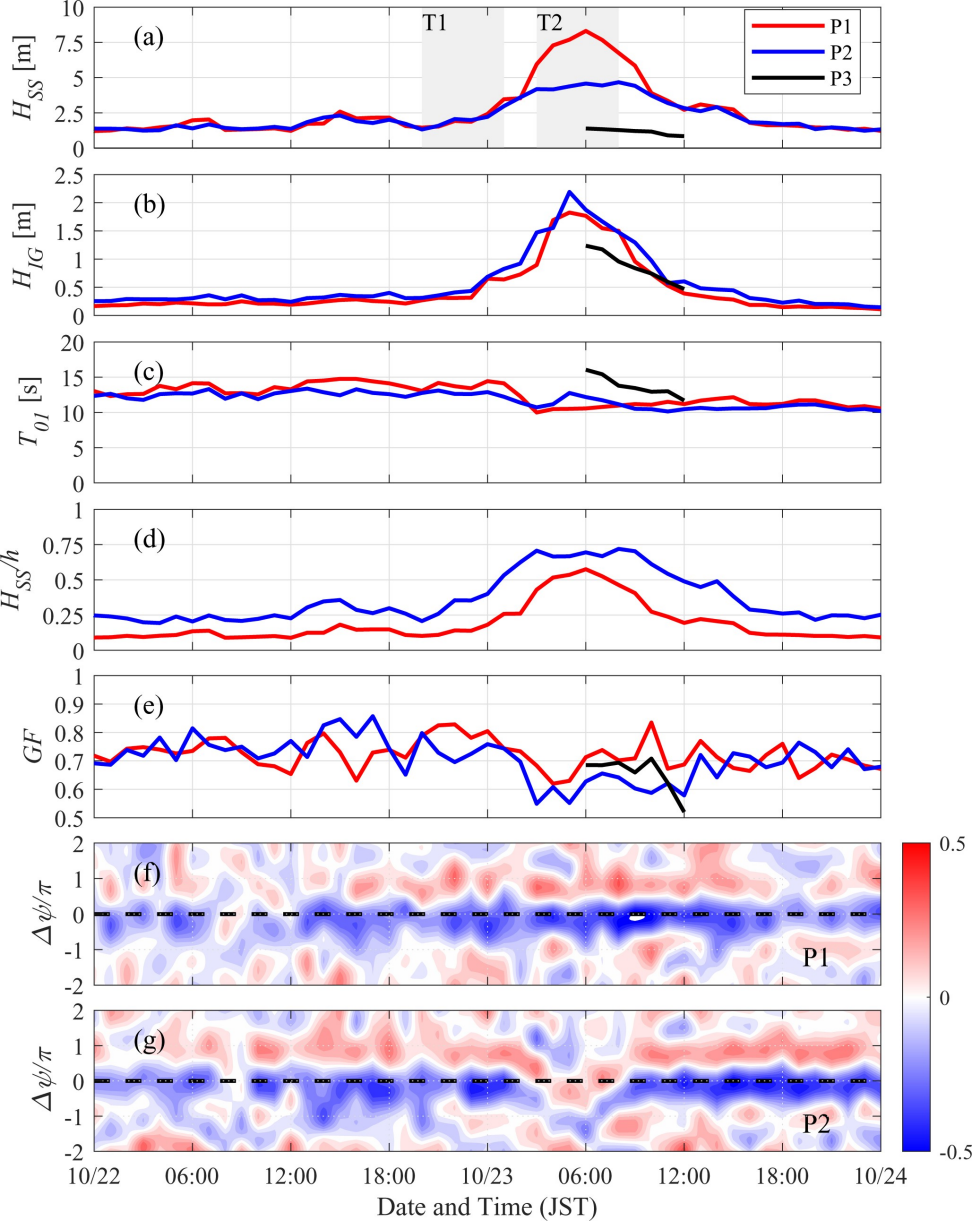
(a) Short wave heights calculated at P1, P2, and P3 (Eq 2). Gray shadings indicate the initial and peak typhoon periods, T1 and T2, respectively. (b) Infragravity-wave heights. (c) Significant wave periods (Eq 3). (d) SS wave-height-to-water-depth ratio. (e) Groupiness factor (Eq 4). (f) and (g) Cross-correlations between the wave-group envelope and IG waves at P1 and P2 (Eq 5). Time lag, ***τ***, is converted to the phase lag of the IG waves by $\Delta \psi =\tau /{T_{01}}_{IG}$.

We calculated the significant wave heights of the SS and IG components and the wave period by using the spectral moments of water surface changes:

$${m_{n}}_{f}=\int_{f}S(f^{\prime }){f^{\prime }}^{n}df^{\prime },$$ (1)

$$H_{f}=4\sqrt{{m_{0}}_{f}},$$ (2)

$${T_{01}}_{f}={m_{0}}_{f}/{m_{1}}_{f},$$ (3)

where *S*(*f*ʹ) is the *fʹ* component (in Hz) of the power spectrum density of water-surface elevation. The frequency-band (*f*) range was 0.04 Hz<*f*_*SS*_<0.20 Hz for the SS components and 0.005 Hz<*f*_*IG*_<0.04 Hz Hz for the IG components, according to values reported in a previous study [17]. The power-spectrum density was calculated with a time window of 128 s and an overlap of 64 s, resulting in a frequency resolution of 0.0078 Hz. Wave characteristics observed during October 22–24 (Fig 2(A)–2(C)) show that short wave height (*H*_*SS*_) reached a maximum value of ~8.3 m at P1 on October 23 at 06:00 JST but decreased to ~4.5 m through wave breaking at P2. Although the along-shore location was offset by 1 km, *H*_*SS*_ decreased to <1.5 m at P3 during peak time. In contrast, infragravity wave height (*H*_*IG*_) at P2 increased to >2 m on October 23 at 05:00 JST. *H*_*IG*_ almost equaled *H*_*SS*_ at P3. For both SS and IG waves, the significant wave period, *T*_01_ was calculated within 0.005 Hz<*f*<0.2 Hz. *T*_01_ increased to ~14 s before but lightly decreased after the October 23 typhoon landfall, indicating the growth of locally generated waves over far-field swells. At P3, *T*_01_ was observed to be >15 s during the peak time, partially because of the dominance of the IG components over SS waves.

The SS wave-height-to-water-depth ratio (Fig 2(D)) was observed to reach a maximum value of ~0.6 at P1 at the peak of the typhoon, after an increase in *H*_*SS*_. In contrast, the ratio reached a value of ~0.7 at P2 on October 23 at 03:00 JST and remained almost constant during the peak period while *H*_*SS*_ at P2 become considerably smaller than *H*_*SS*_ at P1. This clearly indicated that SS wave heights were capped by wave breaking. To investigate the wave grouping degree at each location, we also calculated the groupiness factor (GF), which represents the normalized magnitude of the wave group. We used the method proposed by List [26]:

$$GF=\frac{\sqrt{2}\sigma_{\zeta }}{\langle \zeta (t)\rangle },$$ (4)

where *ζ*(*t*) is the upper envelope of the wave group calculated from band-pass-filtered SS waves, *σ*_*ζ*_ is the standard deviation, and 〈 〉 represents the time averaging. GF ranges from 0 to 1, increasing with the scattering of individual wave heights around the significant wave height. The GF was greater than 0.7 on October 22 at P1 and P2 but decreased to ~0.6 at P2 during the passage of the typhoon (Fig 2(E)). This reduction in GF indicates wave-group collapse because of wave breaking even though the decrease is small when compared with those reported in a previous study on mild-slope beaches [17].

Cross-correlation between the wave-group envelopes *ζ*(*t*) and IG waves *η*_*IG*_(*t*) is a significant indicator for identifying the dominant forcing mechanism of the IG waves. Theoretically, bound waves exhibit troughs below the crests of an upper envelope, thereby defining a phase lag of π in which the upper envelope and IG waves are out of phase. In contrast, the breaking-point-forced IG waves are observed to be in phase with the upper envelope [27,28]. We generated the time series of the cross-correlation coefficient with the hourly phase lag Δ*ψ* between the wave-group envelope *ζ*(*t*) and the IG waves *η*_*IG*_(*t*) at each location (Fig 2(F) and 2(G)). We calculated IG frequency water level change, *η*_*IG*_ by applying a bandpass filter in the IG frequency range. The cross-correlation coefficient *r* can be calculated as

$$r(\tau )=\frac{\langle \zeta (t+\tau )\eta_{IG}(t)\rangle }{\sigma_{\zeta }\sigma_{\eta_{IG}}},$$ (5)

where *τ* is the time lag between *ζ*(*t*) and *η*_*IG*_(*t*). Time lag is converted to phase lag using $\Delta \psi {{=\tau /T}_{01}}_{IG}$ (Fig 2(F) and 2(G)). A negative correlation was observed during the entire period at P1, when Δ*ψ* was ~0, indicating *ζ*(*t*) and *η*_*IG*_(*t*) were out of phase, clearly suggesting that the IG waves developed mainly because of the release of bound waves. Also, the normalized bed-slope parameter [29] *β*<0.3 during the period, further supporting the idea that the dominant mechanism involved is the release of bound waves rather than the breakpoint-forcing mechanism. In contrast, the correlation in the vicinity Δ*ψ* = 0 transformed from a negative to positive value, and the negative peak shifted to Δ*ψ* = −*π* during the peak of the typhoon at P2 inside the surf zone. This shift clearly suggests the reversal of the phase relation between the IG and SS waves by wave breaking.

### 3.2 Individual wave analysis

During the passage of the typhoon, the phase relation between the SS and IG waves exhibited a drastic change at P2 (Fig 2(G)). A similar phase shift was also observed in previous studies [15,17,29–31]. Janssen et al. [31] theoretically demonstrated that the phase lag between forced waves and the wave-group envelope gradually deviated from π on a sloping beach because of the relative delay of the IG waves. They also suggested that a rapid phase shift could be observed in the surf zone because the IG waves modulated the occurrence of SS wave breaking by changing the instantaneous water level. The observed phase shift was mainly attributed to the wave-breaking effect because it occurred only when the P2 was in the surf zone. Although this phase shift has been previously reported, the detailed IG-wave modulating process and its impact on the nearshore wave statistics were not demonstrated based on the *in situ* observation data.

We conducted individual wave analysis to investigate the effect of the IG waves on the nearshore evolution of the SS waves [23,32]. In this study, we estimated the wave height *H* of individual waves by applying the zero down cross method. Then we focused on two representative periods, the initial typhoon period T1 (October 22, 20:00 to October 23, 1:00 JST) when both P1 and P2 were outside the surf zone, and the peak typhoon period T2 (October 23, 3:00–8:00 JST) when P2 was in the surf zone.

To discuss the wave breaking of incident SS waves, we applied the empirical wave-breaking criterion [33,34]:

$$\frac{H_{b}}{h_{b}}=A\frac{L_{0}}{h_{b}}\{1-\exp\left[-1.5\pi \frac{h_{b}}{L_{0}}(1+11s^{\frac{4}{3}})\right]\},$$ (6)

where *H*_*b*_ and *h*_*b*_ are the breaking-wave height and water depth, respectively; *L*_0_ is offshore wavelength (based on significant wave periods); *s* is the bottom slope (*s* = 1/40); and *A* is an empirical constant. Originally *A*<0.17, but 0.12<*A*<0.18 was proposed for irregular waves. The minimum and maximum *A* values represent the lower and upper limits of data scatter, respectively [34]. Eq 6 gives the local maximum ratio of wave height to water depth (as *H*_*b*_/*h*_*b*_) for the given wave period.

**Fig 3:**
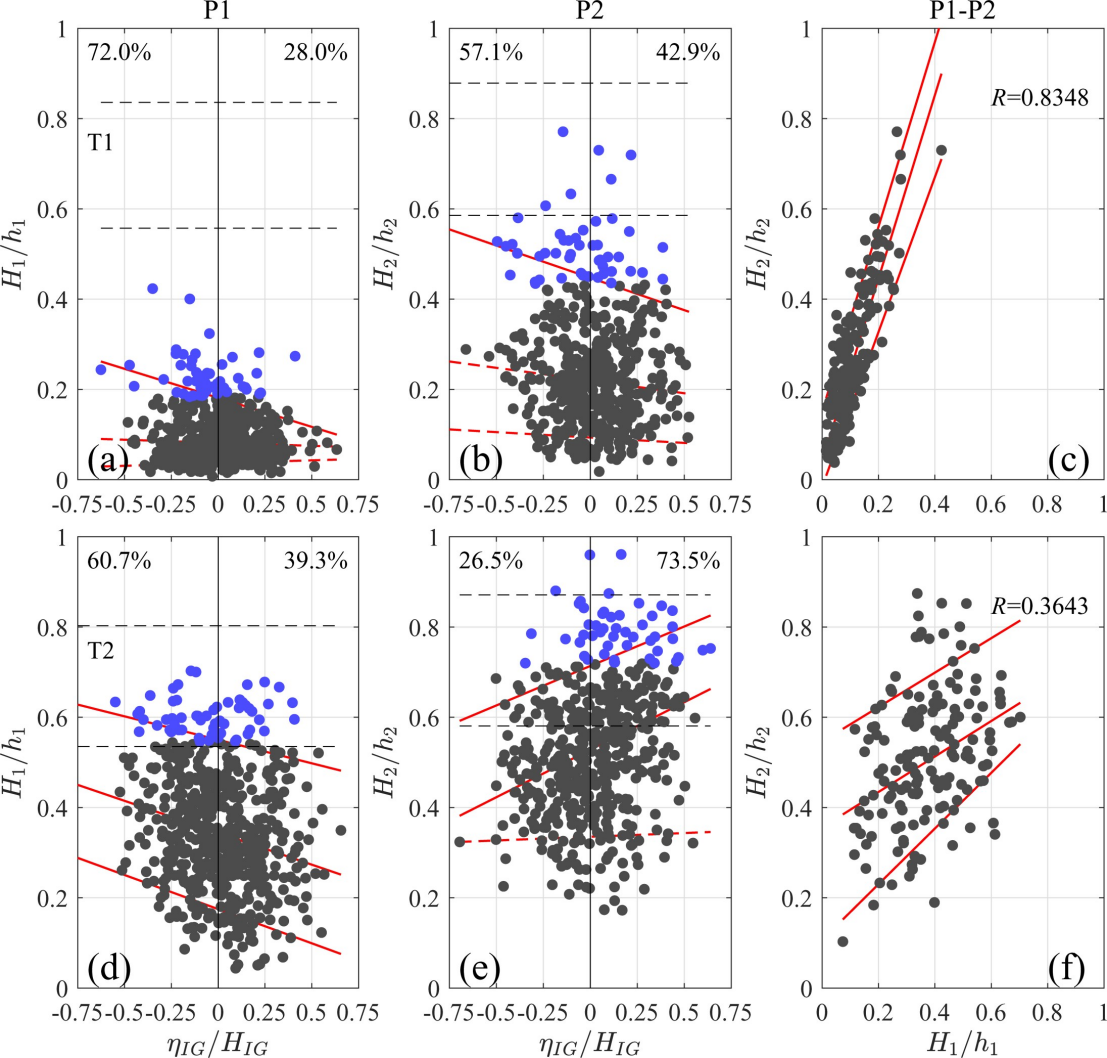
(a) and (b) Relations between individual wave heights and the water-level changes caused by infragravity (IG) waves normalized with wave height (*H*_*IG*_) at locations P1 and P2 during initial typhoon period (T1). Individual-wave heights were normalized with local still water depth. Blue data points correspond to top 10% of waves. Black dashed lines correspond to wave breaking criterion (per Eq 6) for empirical constant *A* = 0.12 and 0.18. Red lines correspond to 10% (lower), 50% (middle), and 90% (upper) quantile regression lines; if slope is not statistically significant, line is dashed. Percentages at top of four graphs indicate bias of top 10% (high) waves between IG-wave troughs and crests. (c) Changes between individual wave heights at P1 and P2 during T1. (d), (e), and (f) Results of the same analyses applied to peak typhoon period (T2).

To assess the effects of the IG water-level change on individual waves, we compared *H*/*h* of individual waves with the normalized instantaneous IG-frequency water level change *η*_*IG*_/*H*_*IG*_ at timings simultaneous with individual wave crests. Accordingly, a negative value of *η*_*IG*_/*H*_*IG*_ indicates that the individual wave was propagating in water of depth shallower than still water depth owing to the IG waves, and vice versa. We calculated the still water depth *h* and the IG-wave height *H*_*IG*_ hourly to minimize the effects of gradual changes in the wave field after the typhoon approach. The scatter plot (Fig 3) of *H*/*h* against *η*_*IG*_/*H*_*IG*_ of each individual wave during T1 and T2 (along with the approximate range of wave-breaking occurrence, Eq 6) includes a regression line for arbitrary 10%, 50%, and 90% quantiles, which can be used to assess the nonuniform variations caused by complex interactions between variables [35]. The robustness was utilized to analyze sand transportation modulated by IG waves in a previous study [36]. The 95% confidence levels were estimated according to Koenker [35].

During T1, high waves (i.e., the top 10% of waves during the period) were observed mostly associated with the IG wave troughs at P1 (72%), representing negative correlation (Fig 2(F)). The *t*-test rejected the null hypothesis that the top 10% waves equally propagated on the IG wave troughs and crests at a 95% confidence level (average of *η*_*IG*_/*H*_*IG*_ = −0.08). Also, the 90% regression line exhibits a negative trend, whereas the 50% and 10% regression lines show a weak trend. High waves were almost equally associated with the IG wave troughs (57%) and crests (43%) at P2, but the 90% regression line exhibited a negative trend. Although some extreme waves associated with the IG-wave troughs were likely to have undergone breaking before arriving at P2, the data points were mostly distributed well below the upper limit of the wave-breaking criterion, suggesting that a significant proportion of waves propagated as non-breaking waves.

To analyze the evolution of individual waves, we tracked the wave-height change from P1 to P2 during a 10-min period in which the two measurements overlapped. We first determined the mean propagation time as the time lag that resulted in the highest correlation between complete wave signals at two locations. Considering the mean propagation time, we matched the individual waves in one signal with those of the nearest crests in another signal. We then eliminated the matching errors by introducing two thresholds defined by differences in wave period of <25% and >50% of the peak wave period. A scatterplot (Fig 3(C)) showing the wave-height changes from P1 to P2 during T1 and the quantile regression lines illustrates the almost linear relation (cross-correlation *R* = 0.83), suggesting the propagation of individual waves as non-breaking waves with shoaling amplification.

Scatterplots and regression lines (Fig 3(D)) suggested that during T2 at P1, the wave-height distribution was partially capped by wave breaking while high waves were biased toward IG-wave troughs. The negative trends shown by the 50% and 10% regression lines can be explained by the dominance of bound over free incident IG waves. In contrast, at P2, the majority (74%) of high waves (top 10%) that are in between the two wave-breaking limit propagated via the IG-wave crests inside the surf zone. The results of the *t*-tests, which supported this observation (average of *η*_*IG*_/*H*_*IG*_ = 0.15), indicated that the IG waves considerably relaxed the breaking limit of individual waves on their crests. Changing the vertical axis from *H*/*h* to *H*/(*h*+*η*_*IG*_) eliminated the bias at P2 during T1 as represented by the change of distribution of high waves (S2 Fig). The results confirmed that the IG waves affected the probability of wave breaking in the surf zone, where their amplitude was significant relative to the still water depth. This indicated that the individual waves retained their energy by propagating via the IG wave crests.

By plotting the evolution of waves from P1 to P2 during T2 (Fig 3(F)), we found that the two locations’ relative wave heights exhibited significant scatter and moderate correlation (*R* = 0.36), unlike that observed during T1. The large waves at P1 became smaller at P2 because of wave breaking, whereas those on the crests of the IG waves at P2 exhibited a moderate magnitude at P1. These results indicated that the extreme nearshore wave heights were not simply determined by the offshore wave heights of the individual waves but were strongly modulated by the IG waves.

Goda’s [37] empirical breaking model and laboratory data suggest that breaking modulation by the IG waves may enhance the maximum wave height by relaxing the wave-height capping. We conducted further analysis to investigate the effects of IG waves on the nearshore wave statistics. The individual waves were divided into three groups according to the corresponding IG-wave phases: crest (*η*_*IG*_>0.15*H*_*IG*_), trough (*η*_*IG*_<−0.15*H*_*IG*_), and middle range (−0.15*H*_*IG*_<*η*_*IG*_<0.15*H*_*IG*_). We compared normalized total-wave histograms and those divided into the three groups by location (P1, P2, and P3) during T2. For quantitative comparison of the histogram shapes, each was subsequently fitted to the Weibull distribution as

$$P(x)=\frac{b}{a}{\left(\frac{x}{a}\right)}^{b-1}\exp\left(-{\left(\frac{x}{a}\right)}^{b}\right),$$ (7)

where *x* is the individual wave height (normalized with the significant wave height *H*_1/3_); *a* is the scale parameter; and *b* is the shape parameter (Note that the Weibull distribution corresponds to the Rayleigh distribution when *b* = 2). We also conducted a Kolmogorov–Smirnov (KS) test to analyze the significance of the histogram differences by locations and by IG wave modulation (The KS test is a nonparametric method to determine whether two datasets differ statistically). We chose to focus only on the differences between two histograms exhibiting a confidence level >95% (p<0.05).

**Fig 4:**
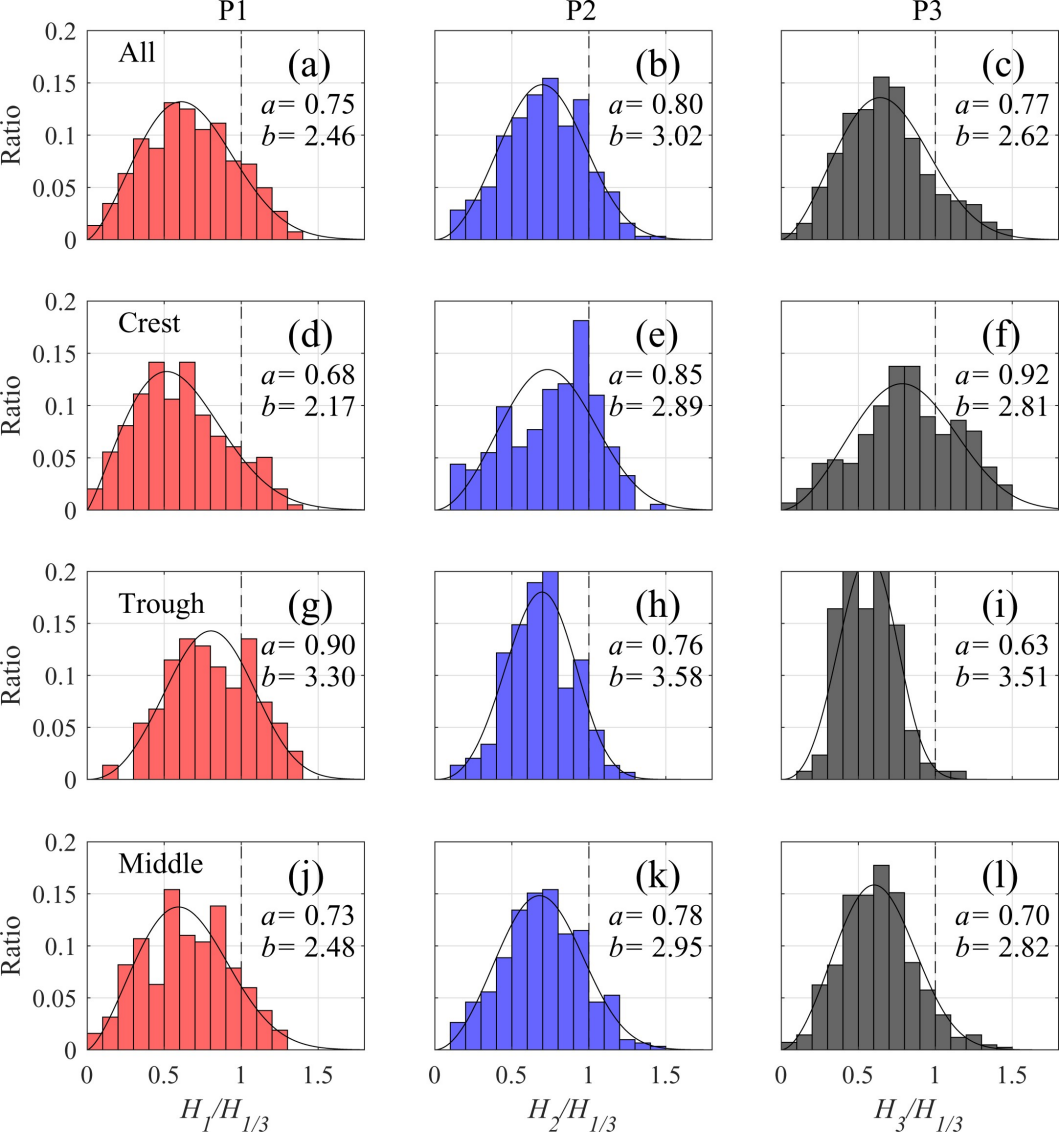
(a)–(c) Normalized wave histograms of total waves during peak typhoon period T2, on the (d)–(f) waves crests, (g)–(i) troughs, and (j)–(l) middles of ranges at observation points P1, P2, and P3. The vertical axis indicates the ratio of the number of the individual waves in each bin to the number of total waves of each group. Solid curves indicate Weibull distributions fitted to each histogram, with values of scale and shape parameters (*a* and *b*) are shown in each graph.

We plotted the observed wave height statistics as histograms and distribution curves (Fig 4). The total histogram at P2 slightly differs from that at P1 (p = 0.003). The increased *a* and *b* values between P1 and P2 indicate positive shifts in the distributions, with sharp peaks at P2. This distribution change can be attributed to the fact that the maximum wave height was strongly limited by the local water depth through wave breaking. Compared to P2, the nearshore waves at P3 exhibit a different distribution (p = 0.006). The decreased *b* value suggests that the histogram acquired a broad-banded distribution, corresponding to the large GF relative to P2 (Fig 2(E)). A similar result was reported from previous field measurements conducted by Inch et al. [17].

The SS waves on the IG wave crests and troughs exhibited different distributions at all the locations (p<0.0001). A distribution peak was observed at P1 on the high-wave side of the troughs, as suggested by *a* (Fig 4(D) and 4(G)). Also, the number of high waves (*H*/*H*_1/3_>1) was considerably greater on the troughs than on the crests. This result confirms previous observations that the IG waves largely propagated as bound waves whose troughs were observed below the crests of the upper envelope of the wave groups. An opposite trend can be observed at P2 with only a small number of high SS waves riding on the troughs (Fig 4(E) and 4(H)). These high waves were considerably susceptible to wave breaking, which also capped their heights. Also, the distribution peak was located at *H*/*H*_1/3_ = 0.9 on the crests with a large number of high SS waves (i.e., larger *a* and smaller *b* values when compared with those on the troughs). The majority of the high SS waves in the total distribution were located on the crests of the IG waves at P2. The shift of the distribution from P1 to P2 can be explained by the wave-breaking modulation of the IG waves. The asymmetry of the histograms between the troughs and crests was remarkably observed at P3 (Fig 4(F) and 4(I)). Although the majority of the waves were smaller than *H*_1/3_ on the troughs, almost half the waves were distributed in the range above *H*_1/3_ with a significant proportion reaching 1.5*H*_1/3_. These step-by-step changes in wave statistics from the offshore to shallow water indicate that the high waves in the surf zone were originally small SS waves in deep water that propagated on the IG-wave crests.

The histograms in the middle range of the IG waves, i.e., those without IG wave modulation, do not show significant differences when compared with the total histograms at P1 (p = 0.82) and P2 (p = 0.69) (Fig 4(J) and 4(K)). This result suggests that the net effect of modulation by the IG waves remained small at these locations. In contrast, the two histograms show significant differences at P3 (p = 0.015, Fig 4(L)). The large *a* and small *b* values of the total histogram indicate that the total histogram exhibits a broader distribution when compared with that in the middle range of the IG waves. This implies that the IG-wave modulation has a residual effect on the wave statistics in the nearshore area, providing opportunities for relatively high waves to reach the shore.

## 4 Discussion and summary

Herein, we presented coastal wave records under the extreme conditions generated by Typhoon Lan in 2017, in which the incident wave heights exceeded 8 m at a depth of 14 m and the IG wave heights reached 2 m in shallow water. The coastal observation of such large IG waves has not been previously documented in literature. The extreme conditions enabled us to study the impact of IG waves on the nearshore dynamics through individual wave analysis. The presence of strong negative correlations between the IG waves and short-wave group outside the breaking zone indicate the dominance of the bound wave mechanism. The energetic IG waves strongly influence the nearshore environment not only in a direct manner but also in an indirect manner by affecting the SS wave characteristics in shallow water. The IG waves appear to modulate the location of SS wave depth-limited breaking by locally reducing (IG trough) or increasing (IG crest) the water depth. The SS waves on the IG wave crests can propagate toward the shore, which potentially leads to strong impacts on the beach and hinterland. This was previously inferred based on laboratory/numerical studies but was not clearly demonstrated in natural beaches. Furthermore, a statistical analysis of the individual waves suggests that the IG waves contributed to the broadening of the wave-height distribution in shallow water, potentially enhancing the extreme impacts of coastal flooding. This study shows that IG waves play a significant role in determining the characteristics of extreme nearshore waves through wave-breaking modulation under extreme conditions. The modulation may not be prominent under moderate conditions when the magnitude of IG waves is negligible compared with the still water depth. This study focused only on the IG wave modulation as instantaneous water level changes, but long-periodic currents by IG waves also can affect SS waves, which could not be discussed with the available data in the present study. Further studies based on detailed measurements and numerical analysis are required to completely elucidate the interactions of SS and IG waves in extreme nearshore environments.

## Supporting information

S1 Fig Time-shifted wave forms at P1 and P2 in accordance with individual wave propagation. Crest nearest to another crest is considered to be its propagated wave crest.
(PS) Click here for additional data file.

S2 Fig (a), (b) Relations between individual wave heights and water-level changes caused by infragravity (IG) waves normalized with wave height (*H*_*IG*_) at locations P1 and P2 during initial typhoon period (T1). Individual wave heights were normalized with local instantaneous water depth modulated by IG waves. Blue data points correspond to top 10% of waves. Black dashed lines correspond to wave breaking criterion (per Eq 6) for empirical constant *A* = 0.12 and 0.18. Red lines correspond to 10% (lower), 50% (middle), and 90% (upper) quantile regression lines; if the slope is not statistically significant, line is dashed. Percentage at top of four graphs indicate bias of top 10% (high) waves between IG-wave troughs and crests. (c) Changes between individual wave heights at P1 and P2 during T1. (d), (e), and (f) results of same analyses applied to peak typhoon period (T2).
(PS) Click here for additional data file.

S1 Appendix Water-surface extraction from video frames. (DOCX)
