# Supplementary material for: Wave-breaking modulation by infragravity waves during an extreme typhoon
Source: PLoS One. 2020 Apr 14;15(4):e0231242. doi: 10.1371/journal.pone.0231242 (PMC7156087; doi:10.1371/journal.pone.0231242)
Supplement: S1 Appendix — (DOCX) [file pone.0231242.s003.docx]

**Appendix S1: Water-surface extraction from video frames**

The brightness with respect to the target array was extracted using every successive video frame, and a time-stack image was generated. The height of the water surface was estimated as the point at which the spatial brightness gradient reached a local maximum near the neighboring estimated water-surface point. The obtained height was based on the image pixel coordinates under the assumption that $\eta=pI+q$, where $I$ is the vertical pixel coordinate, and $p$and $q$ are coefficients that were determined such that the mean water-level changes agree with observed data at the Odawara tide station, which is a few kilo meters west of P3.

(a) Video frame of surveillance camera at P3. Red rectangle outlines target for conducting water-level extractions. (b) Example of time-stack images captured on October 23, 06:00 JST. Red line indicates extracted water levels.
